# Impact of bimonthly repeated total sleep deprivation and recovery sleep on cardiovascular indices

**DOI:** 10.14814/phy2.15841

**Published:** 2023-10-17

**Authors:** Lauren N. Pasetes, Kathleen M. Rosendahl‐Garcia, Namni Goel

**Affiliations:** ^1^ Biological Rhythms Research Laboratory, Department of Psychiatry and Behavioral Sciences Rush University Medical Center Chicago Illinois USA; ^2^ Siemens Healthineers Mountain View California USA

**Keywords:** cardiovascular, echocardiography, recovery, sleep deprivation

## Abstract

Since short sleep duration adversely affects cardiovascular (CV) health, we investigated the effects of exposures to total sleep deprivation (TSD), and baseline (BL) and recovery (REC) sleep on CV measures. We conducted a 5‐day experiment at months 2 and 4 in two separate studies (N = 11 healthy adults; 5 females). During these repeated experiments, CV measures [stroke volume (SV), cardiac index (CI), systemic vascular resistance index (SVRI), left ventricular ejection time, heart rate (HR), systolic and diastolic blood pressure (SBP and DBP) and mean arterial pressure (MAP)] were collected at three assessment time points after: (1) two BL 8 h time‐in‐bed (TIB) sleep opportunity nights; (2) a TSD night; and (3) two REC 8‐10 h TIB nights. CV measures were also collected pre‐study. TSD significantly increased SV and CI, and decreased SVRI, with large effect sizes, which importantly were reversed with recovery, indicating these measures are possible novel biomarkers for assessing the adverse consequences of TSD. Pre‐study SV, CI, SVRI, HR, SBP, and MAP measures also significantly associated with TSD CV responses at months 2 and 4 [Pearson's *r*: 0.615–0.862; *r* ^2^: 0.378–0.743], indicating they are robust correlates of future TSD CV responses. Our novel findings highlight the critical impact of sleep on CV health across time.

## 1 INTRODUCTION

The American Heart Association recently determined that sleep duration is one of the essential eight components for assessing cardiovascular (CV) health (Lloyd‐Jones et al., 2022). Indeed, chronic sleep loss is a serious public health issue associated with CV disease (Cappuccio et al., 2011; Liew & Aung, 2021; Liu & Chen, 2019; Mullington et al., 2009; Tobaldini et al., 2017), as well as numerous other adverse health consequences including cancer, diabetes, Alzheimer's disease, obesity, morbidity, and mortality (Gallicchio & Kalesan, 2009; Mullington et al., 2009; Niu et al., 2022). In response to the two most commonly experienced types of sleep loss, total sleep deprivation (TSD) and sleep restriction, CV measures such as stroke volume (SV), left ventricular ejection time (LVET), systolic blood pressure (SBP) and diastolic blood pressure (DBP), heart rate (HR), cardiac index/cardiac output (CI/CO), systemic vascular resistance index (SVRI), and mean arterial pressure (MAP), have shown inconsistent findings, with some studies reporting alterations, and others showing no changes (Cernych et al., 2021; Kato et al., 2000; Keramidas et al., 2018; Krause et al., 2023; Kuetting et al., 2019; Lü et al., 2018; Mikulski et al., 2006; Muenter et al., 2000; Mullington et al., 2009; Papacocea et al., 2019; Reichenberger et al., 2023; Sauvet et al., 2010; Sunbul et al., 2014; Yamazaki, Rosendahl‐Garcia, et al., 2022; Zhong et al., 2005).

In a highly controlled experiment, we showed that one night of TSD significantly increased SV, LVET, SBP and DBP, but did not significantly alter CI or SVRI (Yamazaki, Rosendahl‐Garcia, et al., 2022). Other groups have reported that one night of TSD produced a significant increase in SBP, DBP, CO, HR, MAP, and SVRI (Cernych et al., 2021; Kato et al., 2000; Krause et al., 2023; Kuetting et al., 2019; Papacocea et al., 2019; Sauvet et al., 2010; Sunbul et al., 2014). By contrast, other research showed that one night of TSD significantly decreased CI/CO, HR, MAP, and SV (Krause et al., 2023; Mikulski et al., 2006; Papacocea et al., 2019; Zhong et al., 2005). Additionally, other studies found that one night of TSD did not significantly change SBP, DBP, MAP, HR, SV, or SVRI (Kato et al., 2000; Mikulski et al., 2006; Zhong et al., 2005). Thus, there are varying results in the literature, and notably, no study has examined the effects of repeated TSD on CV measures across several months.

In addition, our prior study found that SV, LVET, SBP, and DBP returned to baseline (BL) after two nights of recovery (REC) sleep following exposure to acute TSD (Yamazaki, Rosendahl‐Garcia, et al., 2022). To the best of our knowledge, no other studies have examined the effects of subsequent REC sleep on CV measures following a single exposure or repeated exposures to TSD.

The time course of changes in SV, HR, CI, LVET, SBP, DBP, SVRI, and MAP across bimonthly repeated TSD and REC thus far has not been investigated. As such, in this study, we examined changes in these CV measures at 2 and 4 months after repeated exposures to TSD and after REC following TSD in a highly controlled environment. We hypothesized that (1) repeated exposure to TSD would adversely alter CV measures at 2 and 4 months; (2) CV measures would return to BL levels after two nights of REC following exposure to TSD at 2 and 4 months and (3) CV measures assessed pre‐study when fully rested would positively correlate with CV responses during TSD exposures at 2 and 4 months.

## 2 MATERIALS AND METHODS

### 2.1 Participants

Nazemnyy Eksperimental'nyy Kompleks, located in the Institute of Biomedical Problems of the Russian Academy of Sciences, Moscow, Russia, is an isolation facility that accommodates six individuals at a time and is designed to conduct research studies investigating the effects of spaceflight on performance and behavioral health (Anikushina et al., 2022; Cromwell et al., 2021; Fedyay et al., 2023; Le Roy et al., 2023). We conducted a 4‐month study (*N* = 6 healthy adults; 3 females; mean age ± SD, 34.3 ± 5.7 years; mean body mass index ± SD, 22.5 ± 3.2 kg/m^2^) from March 2019–July 2019, and a similar 8‐month study (*N* = 5 healthy adults; 2 females; mean age ± SD, 33.6 ± 5.17 years; mean body mass index ± SD, 27.1 ± 4.9 kg/m^2^) from November 2021–July 2022 in the facility (Anikushina et al., 2022; Bell et al., 2023). Participants had scientific and/or technical backgrounds and human‐support skills relevant for space exploration. Across the two studies, the nationalities of the participants were Russian (*N* = 6), American (*N* = 4), and Emirati (*N* = 1). Participants were screened thoroughly by the National Aeronautics and Space Administration (NASA). Inclusion/exclusion criteria required that the participants originate from various cultures and nationalities, have no prior experience with spaceflight, and include both males and females; all participants were in excellent health—they passed a drug screen, physical exam, and psychological assessment, and had no history of neurological, CV, integumentary, musculoskeletal, or gastrointestinal problems (Abeln et al., 2022; Saveko et al., 2022; Yamazaki, Antler, Casale, et al., 2021; Yamazaki, Rosendahl‐Garcia, et al., 2022). The studies were approved by the Institutional Review Board of the NASA (4‐month study and 8‐month study) with primary oversight, and by the Institutional Review Boards of the University of Pennsylvania (4‐month study) and Rush University Medical Center (8‐month study). All protocol methods were carried out according to approved regulations and guidelines. Participants provided written informed consent prior to inclusion in the study in accordance with the Declaration of Helsinki. Participants received compensation for their participation.

### 2.2 Procedures

**FIGURE 1:**
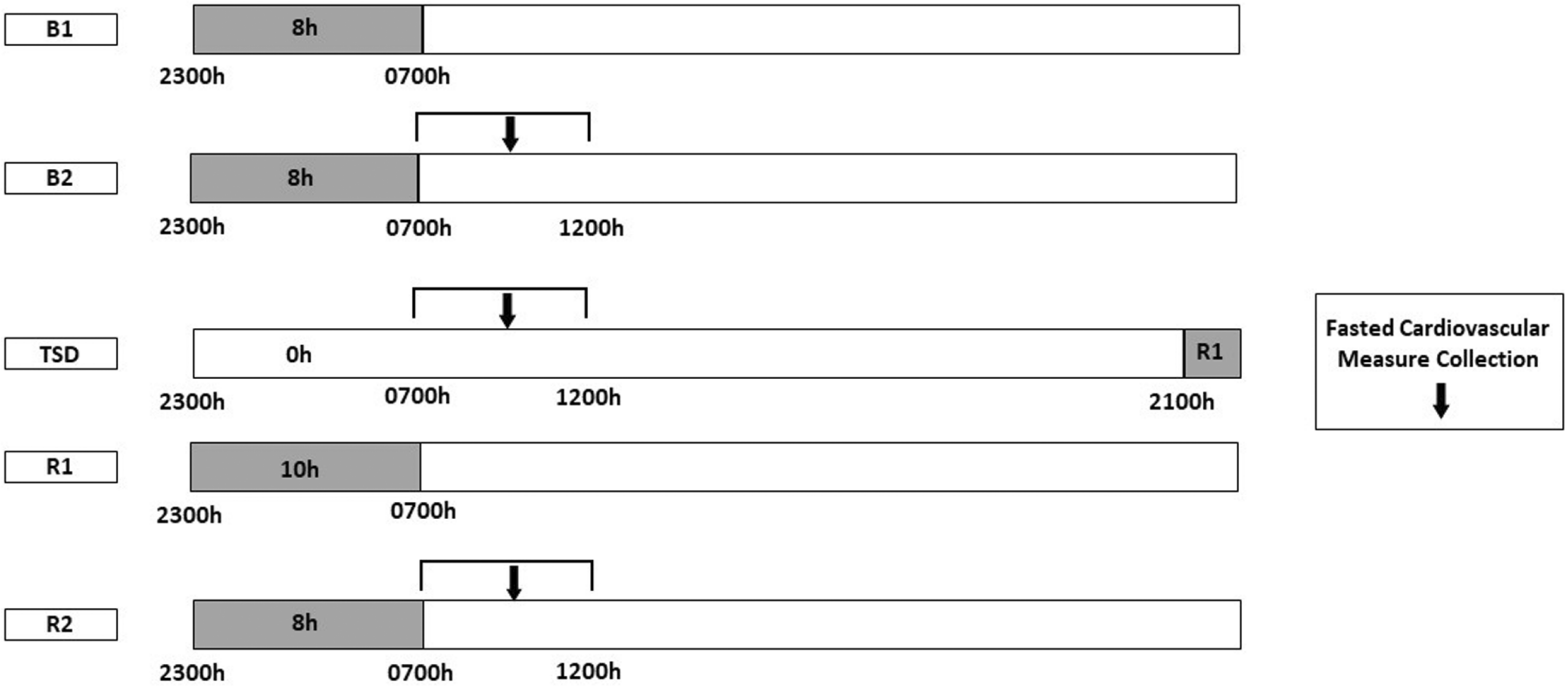
Five‐day experimental protocol. Participants received two nights of baseline with 8 h time in bed (TIB) sleep opportunity (B1, B2; 2300–0700 h). Cardiovascular (CV) measure collection (black arrows) occurred between approximately 0700–1200 h after the B2 night. Following B2 daytime, participants experienced continued wakefulness for approximately 39 h of total sleep deprivation (TSD). During TSD, CV measure collection occurred between approximately 0700–1200 h. Recovery sleep opportunities, which followed approximately 39 h of TSD, were 10 h TIB [(recovery 1 (R1); 2100–0700 h)] and 8 h TIB [(recovery 2 (R2); 2300–0700 h)]. CV measure collection then occurred between approximately 0700–1200 h after the R2 night, during R2 daytime.

During these studies, a five‐day experimental protocol was conducted twice (at months 2 and 4) in the 4‐month study, and a similar experimental protocol was conducted three times (at months 2, 4, and 8) in the 8‐month study (Figure 1). The first two protocols (at months 2 and 4) occurred on the same days in the 4‐month and 8‐month studies. For this paper, we only analyzed data from months 2 and 4 from both studies. Participants received two nights of baseline (BL) with 8 h time in bed (TIB) sleep opportunities [BL 1 (B1), BL 2 (B2); 2300 –0700 h)]. BL CV measure collection occurred between approximately 0700–1200 h after the B2 night. Following B2 daytime, participants experienced continued wakefulness for approximately 39 h of TSD. During nights of acute TSD, participants were ambulatory. They participated in numerous tasks to sustain wakefulness throughout the night during the TSD protocol, such as completing diverse decision‐making and team communications tasks, operational and cognitive performance tasks, as well as surveys and questionnaires. Participants also performed a variety of resupply tasks and routine maintenance in the facility. Participants were monitored continuously by wrist actigraphy and by outside observers to ensure adherence with sustained wakefulness and the protocol during TSD nights. During TSD, CV measure collection occurred between approximately 0700–1200 h. Recovery (REC) sleep opportunities, which followed approximately 39 h of TSD, were 10 h TIB [(REC 1 (R1); 2100–0700 h)] and 8 h TIB [(REC 2 (R2); 2300–0700 h)]. CV measure collection then occurred between approximately 0700–1200 h after the R2 night, during R2 daytime. All assessments were conducted at the same time of day (in the morning before eating). All participants fasted for approximately 9 h or longer prior to all collections to maintain consistency across the studies and among participants. We used this same design, including two nights of BL sleep, 39 h of TSD, and two nights of REC sleep in a similar prior short‐duration study (Yamazaki, Rosendahl‐Garcia, et al., 2022).

Wrist actigraphy (Philips Respironics Healthcare) was used to measure and verify total sleep time (the total amount of time asleep; TST), sleep onset latency (the time it takes to fall asleep; SOL), wake after sleep onset (the amount of time awake after initially falling asleep; WASO), and sleep efficiency (the percentage of time spent asleep out of the total sleep time; SE) during the 5‐day experiments (Table 1). In addition, we examined sleep measures 9–10 days prior to the beginning of these experiments (pre‐experimental phase; Table 1). Actigraphic sleep data were reviewed by two trained investigators (L.N.P. and N.G.) and were assessed as in our previous studies (Brieva et al., 2021; Moreno‐Villanueva et al., 2018; Yamazaki, Antler, Lasek, & Goel, 2021; Yamazaki, Casale, et al., 2022; Yamazaki & Goel, 2020; Yamazaki, Rosendahl‐Garcia, et al., 2022).

**TABLE 1:** Actigraphic sleep data before and during the 5‐day experiments at month 2 and at month 4.

		Month 2	Month 4
		*N* = 11	*N* = 11
Pre‐experimental phase	TST (min)	398.90 ± 32.76^#^	404.71 ± 25.30
	SOL (min)	9.95 ± 9.00^#^	21.69 ± 17.43
	WASO (min)	32.83 ± 15.80^#^	31.20 ± 13.40
	SE (percent)	89.17 ± 2.65^#^	86.94 ± 2.46
Baseline 1	TST (min)	406.77 ± 33.98	421.68 ± 46.31
	SOL (min)	11.07 ± 13.93	9.66 ± 13.61
	WASO (min)	30.41 ± 15.86	27.41 ± 15.64
	SE (percent)	89.48 ± 4.23	90.67 ± 6.14
Baseline 2	TST (min)	398.61 ± 74.51	425.80 ± 44.51
	SOL (min)	6.75 ± 7.59	11.36 ± 15.71
	WASO (min)	29.09 ± 19.02	29.02 ± 13.42
	SE (percent)	91.24 ± 3.06	89.36 ± 5.04
Total sleep deprivation	TST (min)	–	–
	SOL (min)	–	–
	WASO (min)	–	–
	SE (percent)	–	–
Recovery 1	TST (min)	534.36 ± 48.43*	568.18 ± 45.23^†^
	SOL (min)	3.64 ± 5.07	3.68 ± 6.70^†^
	WASO (min)	23.16 ± 14.66	28.90 ± 15.38^†^
	SE (percent)	92.53 ± 2.63	93.84 ± 3.67^†^
Recovery 2	TST (min)	354.39 ± 79.42	397.36 ± 34.90
	SOL (min)	9.68 ± 11.91	9.34 ± 8.27
	WASO (min)	20.32 ± 13.94	26.57 ± 17.67
	SE (percent)	91.06 ± 3.30	91.03 ± 4.16

*Note*: Values are presented as means ± SD. *Month 2 TST was significantly shorter than Month 4 TST as assessed by paired *t*‐test [t (9) = −2.741, *p* = 0.023]; ^#^ *N* = 8; ^†^ *N* = 10.

Abbreviations: SE, sleep efficiency; SOL, sleep onset latency; TST, total sleep time; WASO, wake after sleep onset.

### 2.3 Cardiovascular measure collections

During these repeated experiments, CV indices were collected in a seated position via echocardiography or blood pressure monitor (SBP, DBP, and MAP) at three assessment time points: (1) after two baseline 8 h TIB nights (BL); (2) after a night of acute TSD; and (3) after two recovery nights of 8–10 h TIB (REC). In addition, CV measures were collected pre‐study, which occurred a few days before the beginning of the 4‐month or 8‐month studies. SV, CI, SVRI, SBP, LVET, HR, DBP, and MAP were collected under highly controlled and fasted conditions at all time points including pre‐study, with collections completed between 0700 and 1200 h. Notably, all CV measures were within the ranges reported for healthy adults (Cattermole et al., 2017; Klabunde, 2012; Shaffer & Ginsberg, 2017; Yamazaki, Rosendahl‐Garcia, et al., 2022).

### 2.4 Echocardiogram procedures

Due to strict isolation conditions in the facility, one participant obtained all cardiac ultrasound images on the other four or five participants during each study, and a second participant obtained all cardiac ultrasound images on the primary collector during each study. All ultrasound operators were thoroughly trained to collect ultrasound images and Doppler prior to the study and repeated identical collection procedures across each time point (Yamazaki, Rosendahl‐Garcia, et al., 2022).

SV was collected via ultrasound imaging (GE Vivid q ultrasound system [General Electric Medical Systems, Milwaukee]) in a seated posture at all time points (Arbeille & Herault, 1998; Haites et al., 1985; Ihlen et al., 1984; McLennan et al., 1986; Yamazaki, Rosendahl‐Garcia, et al., 2022). Two‐dimensional images of the left ventricular outflow tract (LVOT) were collected from each participant using a 5S‐RS transducer (Yamazaki, Rosendahl‐Garcia, et al., 2022). The LVOT was imaged from the parasternal long‐axis view while the participants were semi‐supine in a left lateral decubitus posture (Yamazaki, Rosendahl‐Garcia, et al., 2022). Three to four, two‐second cine‐loops of dynamic motion of the LVOT were digitally saved. SV was collected utilizing a continuous wave pencil (Pedof) probe for Doppler interrogation (Yamazaki, Rosendahl‐Garcia, et al., 2022). Continuous wave Doppler signals were taken from the ascending aorta at the suprasternal notch in a seated posture (Yamazaki, Rosendahl‐Garcia, et al., 2022). Three five‐second cine‐loop sweeps of continuous wave Doppler data were collected and digitally stored as proprietary raw data (Yamazaki, Rosendahl‐Garcia, et al., 2022).

A professional sonographer (K.M.R‐G) conducted formal analyses of the echocardiography data. Analysis of the digital data was performed using Echo PAC PC (BT12) software (General Electric Medical Systems). LVOT diameters were measured just proximal to the aortic valve leaflet insertion from three consecutive cine‐loops at the maximum opening of the aortic valve. Five consecutive continuous wave Doppler waveform profiles were traced to calculate the velocity time integral (VTI). The interval between each maximum peak on the Doppler spectral from the ascending aorta was used to calculate HR. The duration of each beat was measured to determine LVET for each SV. The VTI and LVET were then transferred from the Echo PAC software to Excel to calculate SV, HR, and CI using the following formulas:

$$\mathrm{SV}={\left(\text{LVOT cross sectional area}\right)}^{*}\mathrm{VTI}$$

$$\mathrm{CI}=\left(\left({\mathrm{SV}}^{*}\mathrm{HR}\right)/1000\right)/\text{body surface area}$$

Any further continuous wave Doppler waveforms not included in the consecutive SV analysis were analyzed for HR in a seated posture where available.

### 2.5 Blood pressure and SVRI

SBP and DBP were recorded using an Omron BP791IT 10 series Plus Automatic Blood Pressure Monitor with ComFitTM Cuff (Lake Forest) in a seated position on the non‐dominant arm (Yamazaki, Rosendahl‐Garcia, et al., 2022). Participants were seated for three minutes before BP collection. The average value of three consecutive readings, taken one minute apart, was used for analyses. SVRI was calculated by assuming that central venous pressure was zero and by using the following equations (Klabunde, 2012; Norsk et al., 2015; Yamazaki, Rosendahl‐Garcia, et al., 2022):

$$\text{Mean arterial pressure}=\left(\mathrm{SBP}+2^{*}\mathrm{DBP}\right)/3$$

SVRI=mean arterial pressure/CI

### 2.6 Statistical analyses

All statistical analyses were performed using SPSS v26 (SPSS Inc), with *p* ≤ 0.05 considered statistically significant and all statistical tests were two‐tailed. Prior studies have found normal distributions for the CV measures examined in these 4‐month and 8‐month studies (Orme et al., 1999; Yamazaki, Rosendahl‐Garcia, et al., 2022), and other research studies have implemented appropriate statistical tests, such as analysis of variance (ANOVAs) (Kato et al., 2000; Lü et al., 2018; Muenter et al., 2000; Sunbul et al., 2014; Yamazaki, Rosendahl‐Garcia, et al., 2022). Sphericity Assumed corrections for degrees of freedom were applied for all repeated measures (RM) ANOVAs since Mauchly's test was never violated for significant within‐subject effects.

RMANOVAs with “condition” as a within‐subjects factor (e.g., SV at BL, TSD, and REC for month 2 and SV at BL, TSD, and REC for month 4) and “group” as a between‐subjects factor (4‐month study participants vs. 8‐month study participants) determined whether there were differences between the 4‐month and the 8‐month study participant groups in CV measures (SV, HR, CI, SVRI, SBP, LVET, DBP, and MAP) across time points. Data did not significantly differ between the studies and therefore were pooled together for analysis across months 2 and 4 in the combined sample (*N* = 11). Since there were no “group” effects or “condition” by “group” interactions, we conducted RMANOVAs to evaluate CV measures in all participants across “overall condition” (the average of BL at months 2 and 4, the average of TSD at months 2 and 4, and the average of REC at months 2 and 4) as well as across “condition at month 2” (e.g., SV at BL month 2 vs. SV at TSD month 2 vs. SV at REC month 2) and “condition at month 4” (e.g., SV at BL month 4 vs. SV at TSD month 4 vs. SV at REC month 4) separately. Post hoc analyses with Bonferroni corrections compared each condition when there was a significant “overall condition” effect (e.g., the average of SV BL at months 2 and 4 vs. the average of SV TSD at months 2 and 4), a significant “condition at month 2” effect (e.g., SV at BL month 2 vs. SV at TSD month 2), or a significant “condition at month 4” effect (e.g., SV at BL month 4 vs. SV at TSD month 4). Bonferroni‐corrected p‐values are reported. Furthermore, partial eta‐squared (η_p_^2^) was calculated as an estimate of effect size, with the following ranges: 0.01–0.06 (small); 0.06–0.14 (medium); and above 0.14 (large). Data are presented as box and whisker plots with upper and lower whiskers representing the 75th and 25th percentiles. We also investigated relationships between pre‐study CV measures, taken when fully rested, and CV responses to acute TSD at months 2 and 4 using Pearson's correlation coefficient (*r*) and Pearson's R‐squared (*r* ^*2*^; coefficient of determination).

Paired *t*‐tests assessed differences in actigraphic TST, SOL, WASO, and SE for each BL and each REC night between months 2 and 4 (e.g., SOL during B1 at month 2 vs. SOL during B1 at month 4) in the combined sample (*N* = 11). In addition, paired *t*‐tests assessed differences in actigraphic TST, SOL, WASO, and SE for the pre‐experimental phase (9–10 days before B1) between month 2 and month 4 (*N* = 8). The one significant actigraphic sleep measure difference has been noted in Table 1.

## 3 RESULTS

### 3.1 Cardiovascular changes across the 5‐day experiments

#### 3.1.1 SV

**FIGURE 2:**
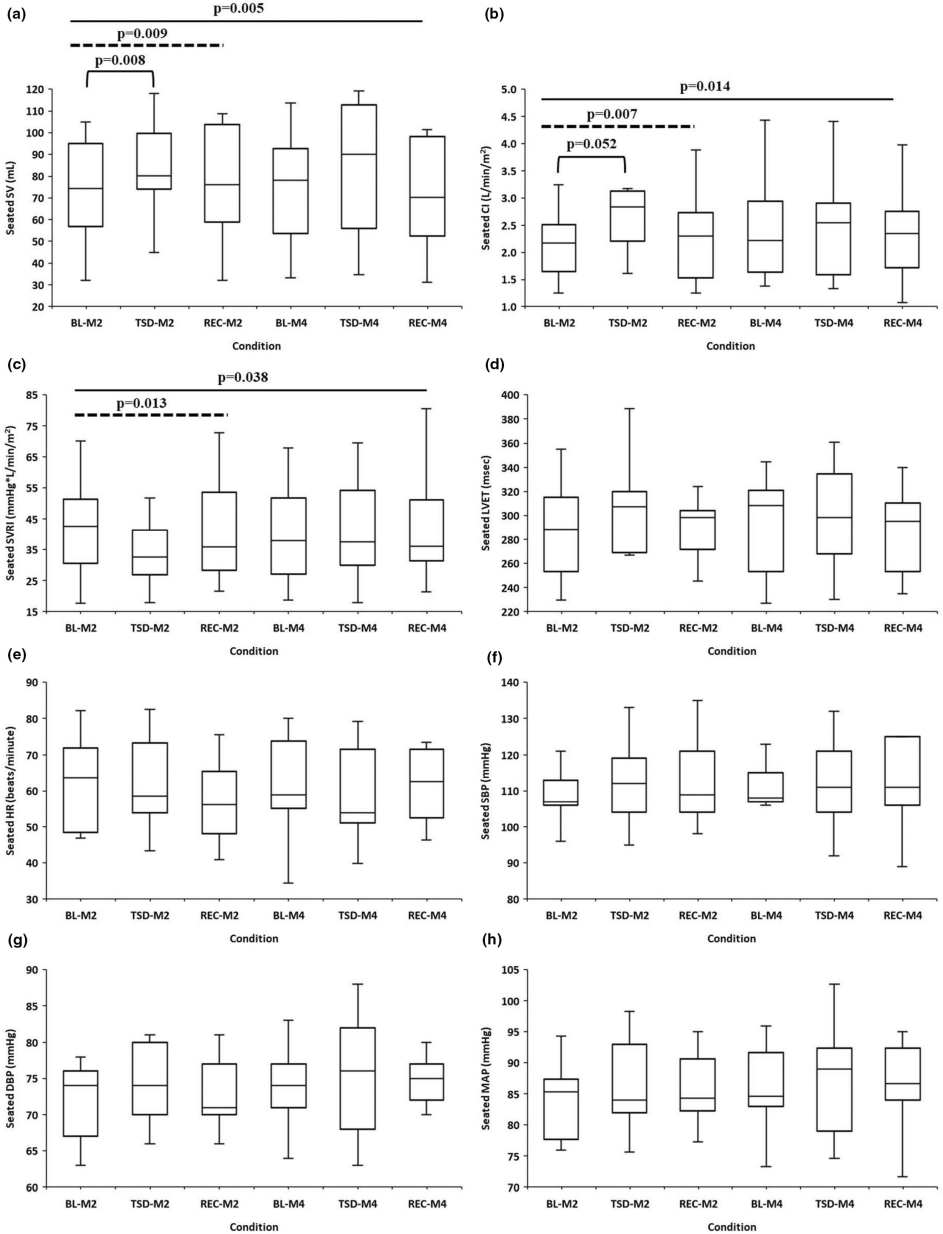
Box and whisker plots indicate changes in seated cardiovascular measures in all participants (*N* = 11) across the 5‐day experiments at months 2 and 4. (a) Stroke volume (SV) demonstrated a significant “overall condition” effect and a significant “condition at month 2” effect, with large estimates of effect size: post hoc analyses with Bonferroni corrections showed TSD SV was significantly greater than BL SV at month 2. (b) Cardiac index (CI) demonstrated a significant “overall condition” effect and a significant “condition at month 2” effect with large estimates of effect size: post hoc analyses with Bonferroni corrections showed TSD CI was significantly greater than BL CI at month 2. (c) Systemic ventricular resistance index (SVRI) demonstrated a significant “overall condition” effect and a significant “condition at month 2” effect, with large estimates of effect size from RMANOVAs. There were no significant “overall condition”, “condition at month 2”, or “condition at month 4” effects from RMANOVAs for (d) left ventricular ejection time (LVET), (e) heart rate (HR), (f) systolic blood pressure (SBP), (g) diastolic blood pressure (DBP), or (h) mean arterial pressure (MAP). In (a–c), solid lines across all time points indicate significant “overall condition” effects from RMANOVAs. In (a–c), dashed lines across BL‐M2, TSD‐M2, and REC‐M2 denote significant “condition at month 2” effects from RMANOVAs. In (a, b), brackets indicate significant post hoc Bonferroni‐corrected comparisons for month 2 time points. In (a–c), *p* values are noted above the lines. Data are presented as box and whisker plots with upper and lower whiskers representing the 75th and 25th percentiles.

Seated SV demonstrated a significant “overall condition” effect, with a large estimate of effect size [F (2, 20) = 6.948, *p* = 0.005, η_p_^2^ = 0.410]: post hoc analyses with Bonferroni corrections showed that TSD SV was significantly greater than BL SV (*p* = 0.026) and REC SV (*p* = 0.049). Seated SV also demonstrated a significant “condition at month 2” effect, with a large estimate of effect size [F (2, 20) = 6.070, *p* = 0.009, η_p_^2^ = 0.378]: post hoc analyses with Bonferroni corrections showed that TSD SV was significantly greater than BL SV (*p* = 0.008; Figure 2a). There was no significant “condition at month 4” effect.

#### 3.1.2 CI

Seated CI demonstrated a significant “overall condition” effect, with a large estimate of effect size [F (2, 20)=5.379, *p* = 0.014, η_p_^2^ = 0.350]: post hoc analyses with Bonferroni corrections showed that TSD CI was significantly greater than REC CI (*p* = 0.022). Seated CI also demonstrated a significant “condition at month 2” effect, with a large estimate of effect size [F (2, 20) = 6.425, *p* = 0.007, η_p_^2^ = 0.391]: post hoc analyses with Bonferroni corrections showed that TSD CI was significantly greater than BL CI (*p* = 0.052; Figure 2b). There was no significant “condition at month 4” effect.

#### 3.1.3 SVRI

Seated SVRI demonstrated a significant “overall condition” effect, with a large estimate of effect size [F (2, 20) = 3.875, p = 0.038, η_p_^2^ = 0.279]. Seated SVRI also showed a significant “condition at month 2” effect, with a large estimate of effect size [F (2, 20) = 5.434, *p* = 0.013, η_p_^2^ = 0.352; Figure 2c]. There was no significant “condition at month 4” effect.

#### 3.1.4 Other CV measures

There were no significant “overall condition”, “condition at month 2”, or “condition at month 4” effects for seated LVET, HR, SBP, DBP, or MAP at month 2 or at month 4 (Figure 2d–h).

#### 3.1.5 Pre‐study CV measures positively correlate with TSD CV responses

We investigated whether pre‐study CV measures correlated with future CV responses to TSD at month 2 and at month 4 (Table 2). Pre‐study SV, CI, SVRI, and SBP all significantly and positively correlated with TSD responses at month 2 and at month 4 (*r*: 0.651–0.862; *r* ^2^: 0.423–0.743, *p* < 0.05). In addition, pre‐study HR significantly and positively correlated with TSD HR at month 4 (*r*: 0.615; *r* ^2^: 0.378, *p* = 0.044), while pre‐study MAP significantly and positively correlated with TSD MAP at month 2 (*r*: 0.639; *r* ^2^: 0.408, *p* = 0.034). By contrast, pre‐study LVET and DBP did not significantly correlate with TSD responses at month 2 or at month 4.

**TABLE 2:** Relationships between pre‐study cardiovascular measures and total sleep deprivation cardiovascular measures at month 2 and at month 4.

CV measure pair	Pearson's *R*	*R* ^2^ value	*p* value (two‐tailed)
SV			
Pre‐study SV and TSD SV (Month 2)	0.651	0.423	**0.030**
Pre‐study SV and TSD SV (Month 4)	0.770	0.593	**0.006**
CI			
Pre‐study CI and TSD CI (Month 2)	0.838	0.703	**0.001**
Pre‐study CI and TSD CI (Month 4)	0.673	0.453	**0.023**
SVRI			
Pre‐study SVRI and TSD SVRI (Month 2)	0.860	0.739	**0.001**
Pre‐study SVRI and TSD SVRI (Month 4)	0.674	0.454	**0.023**
LVET			
Pre‐study LVET and TSD LVET (Month 2)	−0.057	0.003	0.869
Pre‐study LVET and TSD LVET (Month 4)	0.223	0.050	0.510
HR			
Pre‐study HR and TSD HR (Month 2)	0.581	0.338	0.061
Pre‐study HR and TSD HR (Month 4)	0.615	0.378	**0.044**
SBP			
Pre‐study SBP and TSD SBP (Month 2)	0.862	0.743	**0.001**
Pre‐study SBP and TSD SBP (Month 4)	0.800	0.640	**0.003**
DBP			
Pre‐study DBP and TSD DBP (Month 2)	0.462	0.213	0.153
Pre‐study DBP and TSD DBP (Month 4)	0.123	0.015	0.719
MAP			
Pre‐study MAP and TSD MAP (Month 2)	0.639	0.408	**0.034**
Pre‐study MAP and TSD MAP (Month 4)	0.411	0.169	0.209

*Note*: Boldface indicates significant *p* values at *p* < 0.05.

Abbreviations: CI, cardiac index; CV, cardiovascular; DBP, diastolic blood pressure; HR, heart rate; LVET, left ventricular ejection time; MAP, mean arterial pressure; TSD, total sleep deprivation; SBP, systolic blood pressure; SV, stroke volume; SVRI, systemic vascular resistance index.

We also examined whether pre‐study CV measures correlated with future CV responses to TSD using data from *N* = 32 participants in a similar 5‐day experiment conducted within 14–30 day studies in a different NASA isolation facility (Yamazaki, Rosendahl‐Garcia, et al., 2022). As in our current studies, pre‐study SV, CI, SVRI, SBP, HR and MAP all significantly and positively correlated with TSD responses (*r*: 0.537–0.917; *r* ^2^: 0.288–0.840, *p* < 0.01). In addition, pre‐study LVET and DBP significantly and positively correlated with TSD responses in this sample (*r*: 0.551–0.697; *r* ^2^: 0.303–0.485, *p* < 0.01).

## 4 DISCUSSION

Our results showed TSD significantly increased SV and CI and decreased SVRI, all with large estimates of effect size. Furthermore, SV, CI, and SVRI changes were reversed with two nights of REC sleep. Additionally, pre‐study CV measures significantly and positively correlated with future TSD responses for SV, CI, SVRI, SBP, HR, and MAP. Notably, altered CV indices returned to BL levels with two nights of REC sleep, and pre‐study CV measures were robustly associated with TSD CV responses months later. For the first time, we demonstrate that SV, CI, and SVRI are correlates and possible biomarkers for assessing the adverse effects of bimonthly repeated TSD on CV indices in healthy adults. Our results emphasize the significance of adequate sleep duration for CV health, particularly across time.

We found that TSD produced a significant increase in SV, which was consistent with our previous study that had a similar design and CV collection method (Yamazaki, Rosendahl‐Garcia, et al., 2022). We also found TSD significantly increased CI and decreased SVRI, but did not significantly alter LVET, SBP, DBP, HR or MAP. By contrast, our prior study found that one night of TSD significantly increased LVET, SBP, and DBP but did not produce any significant alterations in CI or SVRI (Yamazaki, Rosendahl‐Garcia, et al., 2022). Differences between the current study and our prior study (Yamazaki, Rosendahl‐Garcia, et al., 2022) may be due to that study's shorter duration, single exposure to TSD, or different environmental study conditions, among other factors. Our results were consistent with some prior studies from other groups (Kato et al., 2000; Sunbul et al., 2014; Zhong et al., 2005), but not others (Cernych et al., 2021; Kato et al., 2000; Krause et al., 2023; Kuetting et al., 2019; Mikulski et al., 2006; Papacocea et al., 2019; Sauvet et al., 2010; Sunbul et al., 2014; Zhong et al., 2005). Discrepancies between our results and the findings from other research groups may be due to the shorter durations used in those studies, differing CV collection methods used, or the use of other additional experimental factors such as exercise or caffeine consumption, which can substantially affect CV measures.

Our results showed significant CV changes at month 2 but not at month 4, suggesting a possible CV adaptation to the adverse impact of TSD. In addition, in contrast to month 4, B1 and B2 sleep duration was slightly less than seven hours at month 2, suggesting slight sleep insufficiency, which could possibly explain why there were significant CV alterations at month 2 but not at month 4. However, we note that sleep duration during the pre‐experimental phase before B1 at both months 2 and 4 also was slightly less than seven hours. Future studies should be conducted to examine the repeated effects of TSD on CV indices across longer time intervals in healthy adults.

Notably, some studies have examined sleep loss in adult populations with CV disease. Heart failure patients have been found to show disrupted sleep, reduced sleep duration, and a high risk for obstructive sleep apnea syndromes (Arzt et al., 2006; Pak et al., 2019). Sleep fragmentation has been also associated with congestive heart failure incidence (Yan et al., 2021). In addition, one study found that one night of TSD decreased HR in pre‐hypertensive males and decreased SBP and DBP in both pre‐hypertensive and hypertensive males (Słomko et al., 2018). Future studies should examine the effects of repeated sleep deprivation and subsequent REC in these populations.

Two nights of REC sleep after TSD returned SV, CI, and SVRI to BL levels at month 2, which was consistent with our prior study for SV and CI (Yamazaki, Rosendahl‐Garcia, et al., 2022). Notably, there have been no other studies that have investigated the effects of REC sleep on CV indices following TSD. Beyond CV measures, numerous studies have shown that REC sleep after TSD reduced fatigue and sleep propensity, improved neurobehavioral performance, enhanced mood, and returned C‐reactive protein, delta power, and brain age to BL levels (Casale et al., 2023; Casale & Goel, 2021; Chu et al., 2023; Drummond et al., 2006; Goldschmied et al., 2023; Mantua et al., 2020; Stroemel‐Scheder et al., 2020; Yamazaki, Antler, Casale, et al., 2021; Yamazaki, Antler, Lasek, & Goel, 2021). In contrast, REC sleep did not completely reverse the detrimental effects of sleep deprivation on cortisol levels, behavioral attention, and self‐rated vigor levels (Casale et al., 2023; Casale & Goel, 2021; Yamazaki, Antler, Casale, et al., 2021; Yamazaki, Antler, Lasek, & Goel, 2021). Discrepancies in how effectively REC sleep restores functions to BL levels may be due to the number of REC sleep days employed, variations in the amount of TIB utilized for REC sleep, or interindividual differences, since differential vulnerability to TSD can affect the amount of REC sleep needed for some facets of functioning to return to BL levels (Casale et al., 2023; Casale & Goel, 2021; Yamazaki, Antler, Lasek, & Goel, 2021). Therefore, it is important to further investigate the extent to which REC sleep can completely restore the adverse effects of TSD on CV measures.

Our findings also demonstrated, for the first time, that pre‐study SV, CI, SVRI, and SBP all significantly and positively correlated with TSD responses at months 2 and 4; pre‐study HR significantly and positively correlated with TSD HR at month 4, and pre‐study MAP significantly and positively correlated with TSD MAP at month 2. Importantly, in a separate set of *N* = 32 participants who participated in a similar 5‐day experiment (Yamazaki, Rosendahl‐Garcia, et al., 2022), we also found the same positive, significant relationships for these CV measures. Therefore, several pre‐study CV measures, taken when fully rested, are strong correlates for TSD CV responses months later. Another study (Norsk et al., 2015) found that SV increased with long‐duration spaceflight, as was observed in our current experiments, thus demonstrating the potential application of predicting the adverse effects of sleep loss using CV measures in long‐duration space missions, which have been shown to consistently involve sleep deprivation (Barger et al., 2014; Cromwell et al., 2021; Mhatre et al., 2021).

Our studies have a few limitations. One limitation was the small sample size, which prevented systematic examination of sex or body mass index differences in CV responses to sleep deprivation and REC sleep. In addition, it is difficult to generalize our results to individuals with sleep or mood disorders, or with other medical conditions since our participants were healthy and thoroughly screened. Since the CV indices were assessed in the morning across an approximately five‐hour time frame, there may be a time‐of‐day effect, whereby the results may have been different if taken at another time of day, such as in the evening. In addition, sleep duration was not controlled during the two‐month interval between the 5‐day experiments that occurred at months 2 and 4; however, the sleep indices 9–10 days prior to the 5‐day experiments at both months 2 and 4 were similar to those at B1 and B2. Furthermore, CV collection occurred after two 8 h BL nights before each night of TSD in our protocol to ensure individuals were fully rested and did not have any sleep debt. Another limitation is that our CV measures were collected approximately 24 hours into the 39‐hour TSD time period in order to ensure that all data collection occurred at the same time of day, and therefore our findings may not be representative of more extreme TSD conditions. Our results may also not be generalizable to sleep restriction conditions or to situations that do not involve isolation [our studies were conducted in isolation to simulate long‐duration spaceflight conditions (Fedyay et al., 2023)]. Furthermore, in our studies, we used VTI of the continuous wave form of the ascending aorta as a surrogate measure for LVOT VTI.

In summary, our novel findings demonstrate that SV, CI, and SVRI may be biomarkers for assessing the adverse CV consequences of bimonthly repeated TSD in healthy adults. Notably, altered CV indices returned to BL levels after two nights of REC sleep. Our results also demonstrate that pre‐study CV measures were robustly correlated with future TSD CV responses that occurred months later. Our results underscore the use of CV indices as correlates and possible biomarkers for identification and mitigation of vulnerability in both fully rested and sleep deprivation conditions in the military, and in health and other clinical settings.

## AUTHOR CONTRIBUTIONS

Conceptualization, Namni Goel; methodology, Namni Goel; validation, Namni Goel, Lauren N. Pasetes and Kathleen M. Rosendahl‐Garcia; formal analysis, Lauren N. Pasetes, Kathleen M. Rosendahl‐Garcia, and Namni Goel; writing—review and editing, Namni Goel, Lauren N. Pasetes and Kathleen M. Rosendahl‐Garcia; visualization, Lauren N. Pasetes and Namni Goel; supervision, Namni Goel; project administration, Namni Goel; funding acquisition, Namni Goel; All authors have read and agreed to the submitted version of the manuscript.

## FUNDING INFORMATION

This research was funded by the National Aeronautics and Space Administration (NASA) [grant numbers NNX14AN49G and 80NSSC20K0243 (to N.G.)]. This work was also partially supported by the National Institutes of Health [grant number NIH R01DK117488 (to N.G.)].

## CONFLICT OF INTEREST STATEMENT

The authors declare that the research was conducted in the absence of any commercial or financial relationships that could be construed as a potential conflict of interest.

## Data Availability

The data generated during and/or analyzed during the current study are available from the corresponding author on reasonable request.
